# Nodulation competitiveness and diversification of symbiosis genes in common beans from the American centers of domestication

**DOI:** 10.1038/s41598-022-08720-0

**Published:** 2022-03-17

**Authors:** O. Mario Aguilar, Mónica M. Collavino, Ulises Mancini

**Affiliations:** 1 Instituto de Biotecnología y Biología Molecular (IBBM), Universidad Nacional de La Plata-CONICET, La Plata, Argentina; 2 Instituto de Botánica del Nordeste (IBONE), Facultad de Ciencias Agrarias, Universidad Nacional del Nordeste-CONICET, Corrientes, Argentina

**Keywords:** Ecology, Agroecology

## Abstract

*Phaseolus vulgaris* (common bean), having a proposed Mexican origin within the Americas, comprises three centers of diversification: Mesoamerica, the southern Andes, and the Amotape-Huancabamba Depression in Peru-Ecuador. *Rhizobium etli* is the predominant rhizobium found symbiotically associated with beans in the Americasalthough closely related *Rhizobium* phylotypes have also been detected. To investigate if symbiosis between bean varieties and rhizobia evolved affinity, firstly nodulation competitiveness was studied after inoculation with a mixture of sympatric and allopatric rhizobial strains isolated from the respective geographical regions. Rhizobia strains harboring *nod*C types α and $\upgamma$, which were found predominant in Mexico and Ecuador, were comparable in nodule occupancy at 50% of each in beans from the Mesoamerican and Andean gene pools, but it is one of those two *nod*C types which clearly predominated in Ecuadorian-Peruvian beans as well as in Andean beans *nod*C type $\upgamma$ predominated the sympatric *nod*C type δ. The results indicated that those beans from Ecuador-Peru and Andean region, respectively exhibited no affinity for nodulation by the sympatric rhizobial lineages that were found to be predominant in bean nodules formed in those respective areas. Unlike the strains isolated from Ecuador, *Rhizobium etli* isolated from Mexico as well from the southern Andes was highly competitive for nodulation in beans from Ecuador-Peru, and quite similarly competitive in Mesoamerican and Andean beans. Finally, five gene products associated with symbiosis were examined to analyze variations that could be correlated with nodulation competitiveness. A small GTPase RabA2, transcriptional factors NIN and ASTRAY, and nodulation factor receptors NFR1 and NFR5- indicated high conservation but NIN, NFR1 and NFR5 of beans representative of the Ecuador-Peru genetic pool clustered separated from the Mesoamerican and Andean showing diversification and possible different interaction. These results indicated that both host and bacterial genetics are important for mutual affinity, and that symbiosis is another trait of legumes that could be sensitive to evolutionary influences and local adaptation.

## Introduction

Legumes establish symbiotic associations with the soil bacteria collectively known as rhizobia, which interactions result in the formation of nitrogen-fixing root nodules. The plant specifically recruits the nodule-inducing bacteria that form that novel organ; where they then express nitrogenase, the enzyme that catalyzes the reduction of molecular nitrogen into ammonia. Certain legumes have a broad genotypic range of rhizobia with which they can associate in such a symbiosis^1^.

That *Phaseolus vulgaris*—the common bean, originating in Mesoamerica, with both wild and domesticated forms—has expanded across Latin America, from Mexico down to Northwestern Argentina is universally accepted. Three gene pools have been characterized: two major ones from Mesoamerica and the Southern Andes, and another minor pool with a relatively narrow distribution of wild individuals from the northern Peru-Ecuador area in the Amotape-Huancabamba Depression (AHD)^19,20,26^. The most widely accepted theory states that both the Andean and the northern–Peru-Ecuador gene pools originated through independent migrations from the Mesoamerican populations of Central Mexico. Furthermore, Rendón-Anaya et al*.*^2^ proposed the occurrence of an early dissemination and speciation in Mesoamerica before the split into the two current major gene pools *(i. e.*, Mesoamerican and Andean).

Although the common bean is a promiscuous host, the plant manifests a clear preference for *Rhizobium*, unlike certain closely related *Phaseolus* species that associate with *Bradyrhizobium*^3,4^. Within that bacterial genus, the species *Rhizobium etli* appears to be dominant in common-bean nodules formed in nature in the centers of origin and diversification of the host^5–8^.

In a previous report, we described polymorphism in the common nodulation gene *nod*C among *R. etli* strains from a wide range of geographical origins, which investigation disclosed three *nod*C types. In particular, the different *nod*C alleles in American strains exhibited a varying predominance in their regional distributions in correlation with the centers of bean genetic diversification (BGD centers). The *nod*C alleles α and $\gamma$ were found to be represented equally in isolates of bean nodules as well as in the rhizobial populations retrieved from soil samples in Mexico and Ecuador by using the common bean as a trapping host. However, strains isolated from bean nodules in Ecuador, Colombia, and Brazil were characterized exclusively as $\upgamma$^9^. Furthermore, although both bean rhizobia were significantly retrieved from Ecuadorian soils^9^, a previous survey and a more recent publication on bean-nodule rhizobial isolates in Ecuador reported a novel species, coined *Rhizobium ecuadorense*, that harbor *nod*C type γ and phylogenetically related to *Rhizobium etl*i^10^. In contrast, similar analyses on rhizobia isolated from bean nodules and soil samples of the Andean region indicated a predominance of a *nod*C allele type $\updelta$, whereas *nod*C type γ was not detected. Thus, while the predominance of bean rhizobia containing *nod*C α and $\upgamma$ was detected in Mesoamerica, a dominance of strain $\updelta$ in the soil and nodules from the southern Andes was revealed, whereas a variable balance of α and $\upgamma$ populations were found in the soil and nodules collected in Ecuador-Peru.

Within this context, it is hypothesized that the common bean has an affinity for nodulation by the sympatric rhizobial lineages. Therefore, this work aimed to investigate the symbiosis between the host beans and the nodulating rhizobia representatives of the center of diversification. Allopatric and sympatric bean rhizobial strains previously reported belonging to specific taxonomic units were assessed. Furthermore, analyses of five host genes associated with symbiosis were examined to analyze variations that could be correlated with nodulation competitiveness. The biogeography of the host and its interactions provide a framework for assessing how host mobility affects rhizobial interactions, where the latter can be influenced by conditions such as ecological adaptation, coevolution, and edaphic characteristics.

## Results

### Distribution of rhizobium-symbiosis prevalence among common beans from diversification centers upon coinoculation with a mixture of allopatric and sympatric rhizobia

We examined the nodule occupancy in representative common-bean accessions from the BGD centers from Mesoamerica, the AHD region of Ecuador-Peru, and the southern Andes after inoculation with a mixture of two rhizobial strains. We selected rhizobia strains that represent lineages from the respective geographical regions of bean diversification, which in addition have been genetically investigated and reported to belong to defined taxonomic identities^10,11^ (Table 1).

**Table 1:** Bean rhizobia and plant material.

Strains	*nod*C type	Origin	Source*
*R. etli* CE3	α	Guanajuato, Mexico	S. Brom
*R. etli* SC15	α	Northwestern Argentina	this lab
*R. etli* NOA124	δ	Northwestern Argentina	this lab
*R. ecuadorense* CNPSo 671	γ	Ecuador	M. Hungria
*R. ecuadorense* CNPSo 683	γ	Ecuador	M. Hungria
*R.* sp. CIAT894	γ	Colombia	S. Brom
*R. etli* Bra-5	γ	Brazil	S. Brom
***Phaseolus vulgaris* varieties and/or accessions**			
**Mexico**			
G1820			CIAT
Negro Xamapa			CIAT
**Ecuador**			
G23582			CIAT
G23724			CIAT
**Peru**			
G21244			CIAT
G21245			CIAT
G23587			CIAT
**Andean**			
G19833			CIAT
G19896			CIAT
Alubia Cerrillos			INTA

*CIAT, Centro Internacional de Agricultura Tropical; Cali, Colombia; S. Brom, Centro de Ciencias Genómicas, Cuernavaca, Mexico; M. Hungría, EMBRAPA Soja, Londrina, Brazil; INTA, Estación Experimental Agropecuaria Salta, Salta, Argentina.

All of them were able to induce nodule formation in the bean varieties examined regardless of the plants' genetic pool. These strains represent distinguishable *nod*C-gene genotypes, which characteristic applied here facilitated the identification of a given nodule-occupant strain after bean coinoculation^9^.

**Figure 1:**
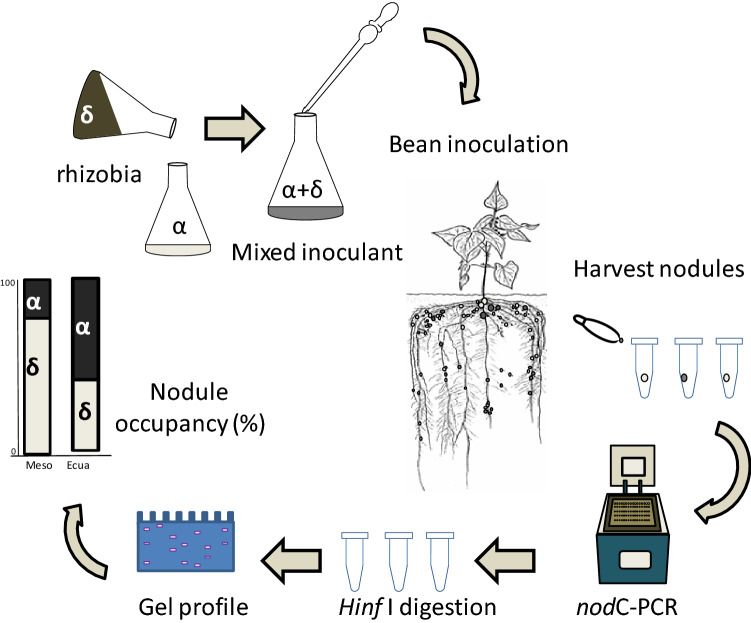
Experimental design to perform competitiveness analysis of nodule occupancy in common beans inoculated with a mixture of sympatric and allopatric rhizobia. Nodules were harvested three weeks after inoculation.

The indigenous strains isolated from Mexico and Northern Argentina harboring the *nod*C type α were assigned to species *R. etli*, whereas their phylogenetically related *R. ecuadorense* strains CNPSo671, CIAT894, and Bra-5, bearing *nod*C type $\upgamma$, had been isolated from Ecuador, Colombia, and Brazil, respectively. The roots of the bean plantlets were inoculated with a two-strain mixture according to an experimental design involving side by side cultivars of representative beans from the Mesoamerican, Andean, and Ecuadorian-Peruvian genetic pools (Fig. 1).

**Figure 2:**
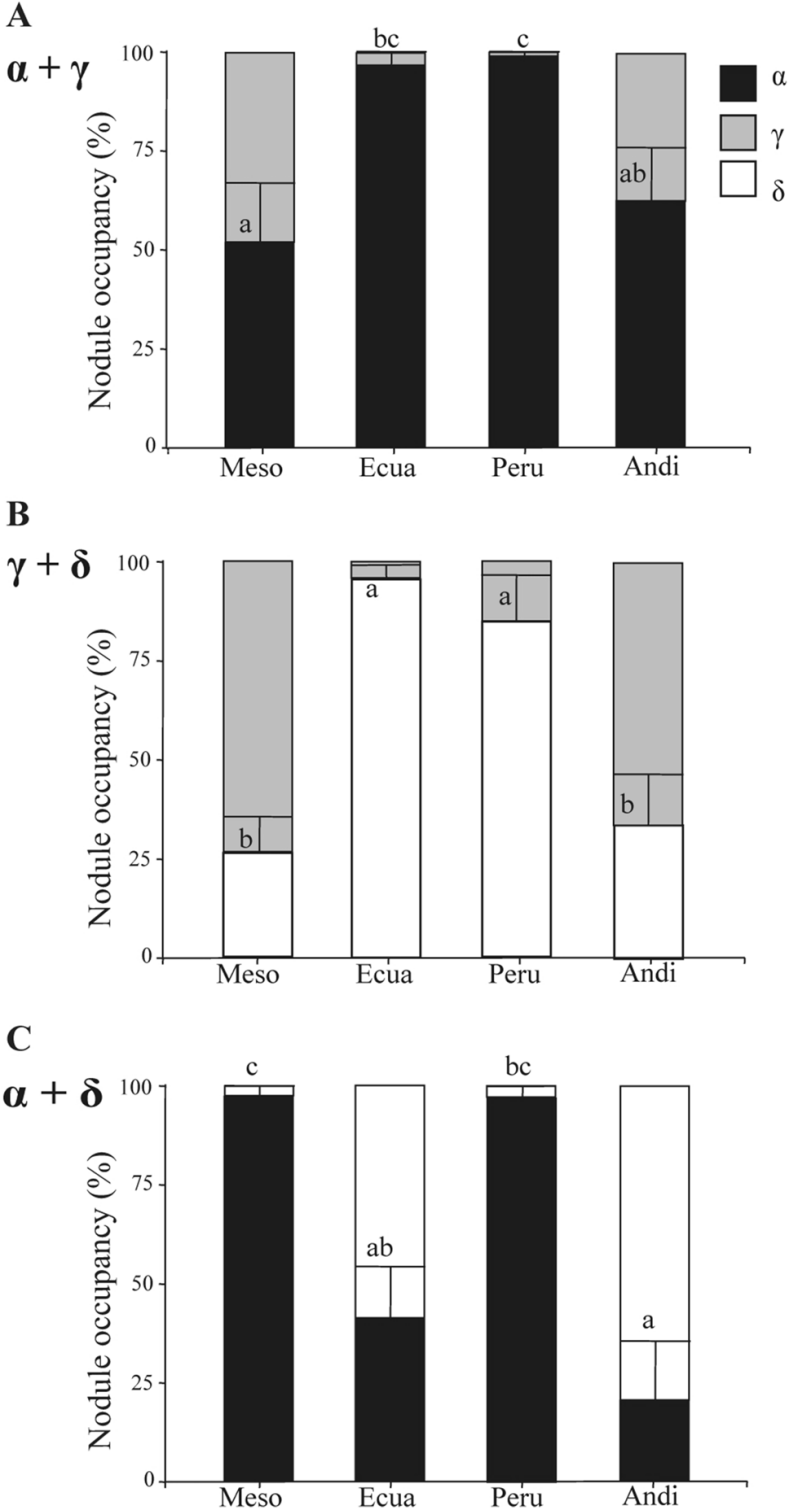
Nodule occupation by *R. etli* strains *nod*C types co-inoculated in bean plants representative of the Mesoamerican (Meso), Ecuadorian (Ecua), Peruvian (Peru), and Andean (Andi) gene pools. Nodule occupancy was defined as the proportion of nodules occupied by each *R. etli* strain *nod*C type, α (black bars), γ (gray bars) or δ (white bars), co-inoculated with a 1: 1 ratio in the following combinations: α with γ (**A**), γ with δ (**B**) and α with δ (**C**). The data are combinations of three independent co-inoculation experiments in which at least two plants of each accession were individually inoculated with a mixture of strains. Statistical analyses were performed with nonparametric Kruskal–Wallis H test. Letters represent groups significantly different (*p* < 0.05) from each other. Bars indicate standard error.

Figure 2 illustrates the frequency of nodule occupancy by one or the other strain each containing a distinguishable *nod*C type. No nodule co-occupancy by both strains was observed. The proportion of nodules occupied by *nod*C type α and γ (Panel A) was comparable, at about 50% each, in the beans from the respective Mesoamerican and Andean gene pools. In nodules formed by Ecuadorian-Peruvian beans, however, the strains featuring the *nod*C type α dominated in occupancy (95 and 85%, respectively) over those of the *nod*C type $\upgamma$ (*p* = 0.0018). This result indicated that, under our experimental conditions, the bean accessions from Ecuador and Peru exhibited no affinity for nodulation by the sympatric rhizobial strains (expressing the *nod*C type $\upgamma$) as compared to those of the *nod*C type α.

Likewise, we assessed the profile of competitiveness between bean rhizobial lineages of the Ecuadorean *nod*C type $\upgamma$ and of the southern-Andean *nod*C type $\updelta$ (Panel B). The relative degree of nodule occupancy revealed that the *nod*C type $\updelta$ was clearly dominant in common beans from the Ecuadorean and Peruvian gene pools (95 and 85%, respectively), whereas a much lower representation of that *nod*C type was significantly detected (*p* = 0.0201) in plants of Mesoamerican and Andean origin, 27% and 33%, respectively. This result indicated that rhizobia containing *nod*C type $\updelta$ competed as well against rhizobia of *nod*C type $\upgamma$ as those bearing *nod*C type α for the nodulation of beans from Ecuador-Peru. In addition to these experiments, we also tested the nodulation after coinoculation with strains containing *nod*C type α and $\updelta$ (Panel C). The results demonstrated that nodule occupancy by *nod*C type α displayed a clear dominance in Mesoamerican and Peruvian plants (98% and 97%, respectively) with a significant occupancy (*p* = 0.0293), as well by *nod*C type δ in the Andean beans (80%). The similar occupancy of both strains observed in the bean from Ecuador indicated no particular preferential affinity for nodulation by either *nod*C strain. Thus, occupancy by rhizobia *nod*C type δ predominated in Andean and Ecuadorean beans, whereas *nod*C type α was significantly high in Mesoamerican and Peruvian plants, which findings confirmed earlier results reported by Aguilar et al.^9^.

Some differential occupation by bean variety was observed comparing the different co-inoculations. The strains *nod*C type $\upgamma$, originally isolated from nodules of Ecuador, displayed a high ratio of occupancy in beans from Mesoamerica and Southern Andes (*p* = 0.0001) as compared with occupancy in bean representatives of the Ecuador-Peru gene pool. Furthermore, *nod*C type α was significantly higher in Peruvian bean than in Mesoamerican and Andean bean varieties (*p* = 0.0032).

Taken together, the results of these co-inoculations testing the competition for nodulation between sympatric and allopatric rhizobia strains revealed no obvious affinity between the different bean pools and their corresponding sympatric strains.

To assess if soil affects competitiveness, an experimental approach was done with two local soils. Joint analysis of nodule occupancy for two contrasting soils of Argentina shared between perlite-substrate and soil showed no significant soil effect as it is referred further on in Discussion.

### Analysis of genes associated with symbiosis in the bean diversity

**Figure 3:**
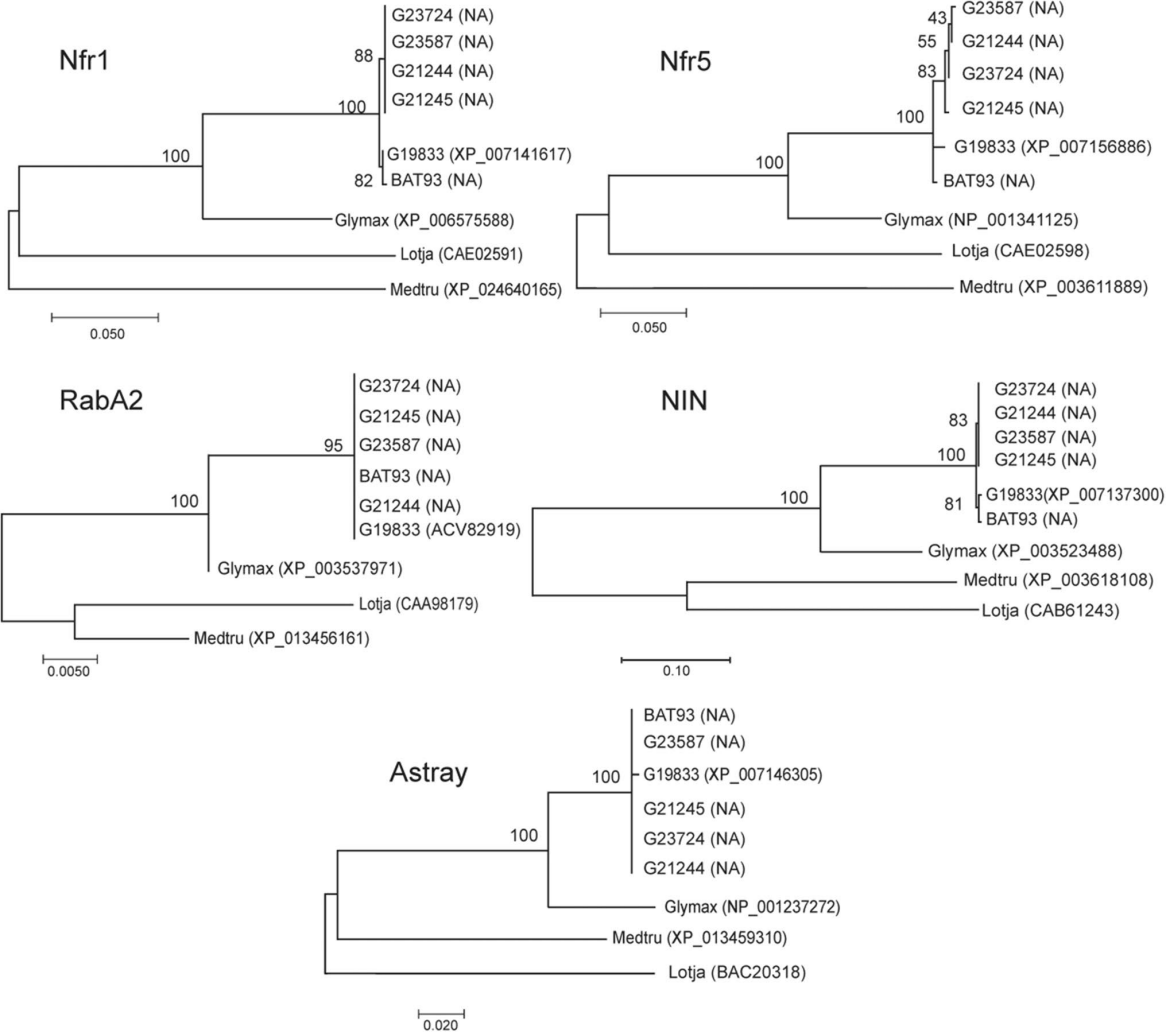
Maximum-likelihood phylogenetic tree of proteins Nfr1; Nfr5; RabA2; NIN and ASTRAY of beans from diversification centers from the Americas. Accessions from: Mesoamerica, BAT93; Ecuador, G23724; Peru, G21244, G21245, G23587; Andes, G19833 and model legumes *M. truncatula* (Medtru), *L. japonicus* (Lotja) and *Glycine max* (Glymax). Identifiers are not assigned (NA) for BAT93 G23587, G23724, G21244 and G21245 proteins.

To assess whether the prevalence of rhizobia reflects variation among the host, a limited number of genes that have been reported to be associated with symbiosis were examined in bean accessions from the BGD. The five bean genes were—RabA2, a small GTPase linked to a major SNP associated with nodulation^12^; ASTRAY, a transcription factor involved in the regulation of nodulation that was found in a QTL for symbiotic nitrogen fixation^13,14^; NIN (*N*odule *In*ception, a transcription factor essential for nodulation^15^ and the host nod-factor—receptor genes NFR1 and NFR5 involved in partner selection within the microsymbiont and early symbiosis-signalling pathways^16^. A release of reference genomes of acceptable quality for the Mesoamerican^17^ and Andean^18^ gene pools as well as for the wild beans from the Ecuadorian-Peruvian AHD regions^2^ enabled us to perform comparative phylogeny among the sequences representative of that diversity as well as with orthologs from the model legumes *Medicago truncatula*, *Lotus japonicus* and *Glycine max*. The genomes of five bean accessions used in the experiments described in the preceding section were examined in this analysis. A comparison of the DNA sequences involving Clustal multiple-sequence alignment is depicted in Supplementary Fig. S1. Sequences of RabA2 and ASTRAY were highly conserved among bean varieties, whereas diverged from that of *L. japonicus* and *M. truncatula* and showed to be close to *G. max*. Contrarily, polymorphism throughout the sequences of NFR1 and NFR5 was identified, and single variations in the amino acids of NFR1 and NFR5 were distinguished as follows: In Mesoamerican and Andean beans and those from the AHD, certain substitutions were not associated with any of the three BGD centers, whereas other mutations were highly conserved in the alleles of the Ecuadorian-Peruvian beans. Thus, NFR1 protein displayed high conservation in the amino terminal and highly conserved amino-acid substitutions in Ecuadorian-Peruvian beans in positions 45 (T- > P), 142 (D- > G), and position 542 (A- > T) in the protein kinase domain; whereas positions in the NFR5 protein are 33 (F- > S), 57 (S- > T), 445 (S- > C), and 577 (A- > G); in general, the LysM domain of NFR5 -positions 51–228- displayed good conservation. Figure 3 presents the phylogenetic tree obtained from the maximum-likelihood method for ASTRAY, RabA2, NFR1 and NFR5 amino-acid sequences. Unlike ASTRAY and RabA2, the NIN, NFR1 and NFR5 proteins place the beans from the Ecuador-Peru area in a cluster separated from that of the beans from Mesoamerica and the Andes (Fig. 3). This result was supported by those obtained after analysis of the model topology of NFR1 and NFR5 polypeptides as in Supplementary Table S2. Indeed, values of the root-mean-square deviation of atomic positions (RMSD) which yield a metric for the evolutionary similarity between proteins, were found similar among bean varieties of the Peruvian-Ecuadorian pool but grouped separately of those of the Mesoamerican and Andean pools (Supplementary Table S2). This result and that presented in Fig. 3 agree with each other.

## Discussion

In the work reported here, we have examined the interaction of symbiotic partners representative of the three major diversification centers. Although *P. vulgaris* could establish symbiosis with diverse rhizobial lineages, *Rhizobium etli* seemed to predominate in nature in the bean nodules collected from the Americas^8,9^, while the Americas is where the origin and diversification of the host have been experimentally supported^19,20^. Genotypes other than *R. etli* that also induce nodule formation in the bean have already been taxonomically defined as species, for instance *Rhizobium tropici* and *Rhizobium ecuadorense*, both of which were isolated from areas in northwestern South America, namely Ecuador, Brazil, and Colombia.

American-bean rhizobia, from soil samples retrieved by the common bean as well as isolates from nodules found in nature have possessed polymorphism in the *nod*C gene, disclosing three *nod*C genotypes namely α, $\upgamma$, and $\updelta$^9^. The different *nod*C alleles in American strains exhibit a varying predominance in their regional distributions in correlation with the centers of bean genetic diversification. The *nod*C types α and $\upgamma$ were detected both in bean nodules and in soils from Mexico, whereas the *nod*C type $\updelta$ was clearly predominant in soil and nodules from the Southern Andes (*i. e.*, in Bolivia and northwest Argentina^9^). A quantitatively balanced representation of rhizobia with *nod*C type α and $\upgamma$ was detected in soils from Ecuador, but the *nod*C type $\upgamma$ had been found to be predominantly isolated from nodules formed in nature in that area^5,9,10^. It should be noted that we have reported finding of equal distribution of allele *nod*C type α and γ among the nine *R etli* isolates from bean in Mexico reported by Silva et al*.*^7,9^. The occurrence of this polymorphism proved to contribute to examining rhizobial populations inhabiting the Americas and characterizing the interaction with beans in BGD centers from Mexico to the northwest of Argentina. In performing our *nod*C analysis, we were aware that rhizobia genes for symbiosis are carried on plasmids which might mediate horizontal transfer, however in agreement with Silva et al*.*^7^ we assumed that although genetic exchange could be important, it is not so extensive to prevent epidemic clones from arising at significant frequency. Similar findings were found in *R. leguminosarum* bv *trifolii* associated with native *Trifolium* species growing in nature^21^.

Investigations in the last decade have proposed an evolutionary pathway for the host bean that provided us with a framework for examining our results on rhizobia-bean interactions and facilitated an interpretation of the results. The current model proposes the occurrence of a Mesoamerican origin from where dispersion by independent migrations over time led to the Mesoamerican and Andean gene pools and to the Ecuador-Peru wild common-bean populations^2,19,20^. We found a balanced competition between α and $\upgamma$ *nod*C types in beans from Mesoamerica and the southern Andes, whereas the beans from Ecuador and Peru revealed a clear affinity for nodulation with strains of *nodC* type α rather than with the sympatric strains *nod*C type $\upgamma$ that we assayed (*R. ecuadorense*, CIAT894 and Bra-5). Nevertheless, we have previously reported that native strains and isolates with respectively both *nod*C types α and $\upgamma$ were found in soils and bean nodules from Mexico^9^, whereas lineages harboring *nod*C type $\upgamma$ were found to be predominant in beans from the northern and central regions of Ecuador-Peru^8,9^. The present results, however, indicated a clear affinity of the Ecuadorean-Peruvian—*i. e.*, AHD—beans for strains *nod*C type α when assessed for competition against *nod*C type $\upgamma$ (Fig. 2A). We also found that *nod*C type $\updelta$ displayed a clear predominant occupancy of nodules of the AHD beans in contrast to the scarce occupancy of nodules of the Mesoamerican and Andean beans (Fig. 2B). Taken together, these results indicate no affinity of AHD beans for sympatric rhizobial strains containing *nod*C type $\upgamma$—despite the finding that rhizobia of *nod*C type $\upgamma$ appear to predominate in isolates of nodules formed in Ecuador^9,10^.

We conclude that although rhizobial type *nod*C $\upgamma$ was previously found to predominate in bean nodules from Ecuador, the competitiveness of that rhizobial strain for nodulation compared to other genotypes of bean rhizobia was relatively low. A possible explanation could be that seeds might be assumed to play a key role as carriers during the dissemination of the bean throughout the American regions. Thus, we can hypothesize that at the time of bean dissemination both *R. etli nod*C types α and $\upgamma$ (*R. ecuadorense* and other lineages) moved in conjunction with the host from Mesoamerica to northern Ecuador-Peru, but the strains bearing *nod*C type $\upgamma$ achieved an adaptation—probably due to edaphic characteristics, environmental stresses, and other features—in such a way that that strain predominated in soils and succeeded in nodulation.

Alternatively, that prevalence might arise from a host selection for a rhizobium that is more effective in nitrogen fixation in a new and different environment. A poor relationship, however, between competitiveness and efficiency was found in the pea^22^. In line with the concept of adaptation, the bean had been found to be preferentially nodulated by species of *R. tropici* in acidic soils in regions of Brazil and Africa^4,23^. Since the environment could also be a major influence on the host and its symbiotic interactions, the Andean area represents a cooler climate for the growth of the bean than the Mesoamerican region^24,25^. Furthermore, since our assays were performed in laboratory environment parameters, we do not rule out the effect -if any- by the diverse and complex soil microbial community coexisting with bean rhizobia. Within this context, two contrasting soils from Argentina which differ in geolocation and edaphic properties and the perlite substrate were assayed side by side in nodule occupancy of Negro Xamapa after inoculation with a mixture of strains *nod*C type α and γ (Results not shown). Our results showed that the predominance of *nod*C type γ in the occupation of the nodules of this variety (about 80% occupation) is not affected by the type of substrate (*p* = 0.5566). Yet, we assume that the performance in diverse soil and ecosystems should be further evaluated in situ. In agreement, a good coevolution of rhizobia strains with *nod*C type $\upgamma$ was detected in nodules of bean varieties from the Mesoamerica and Andean genetic pools inoculated with soil samples from Mexico, Ecuador, and Northwest of Argentina, respectively (see Table 2 in Aguilar et al., 2004) [^9^].

With respect to the interaction in the southern Andes, we propose another interpretation that takes into consideration the bottleneck that occurred before domestication in the Andes, as was indicated by Bitocchi et al*.*^26^, which scenario enables the assumption that the adaptation and concomitant diversification involved a coevolution of the symbioses. Therefore, similar profiles of competitiveness for nodulation in Mesoamerican and Andean beans were found between *nod*C type $\upgamma$ versus *nod*C types α and $\updelta$, but a significant occupancy by the *nod*C type $\updelta$ was recorded in the Andean beans.

Our work suggests that the genetics of both the host and the bacteria determine the mutual affinity and additionally indicates that symbiotic interaction is another trait of legumes sensitive to the effects of evolution and ecological adaptation to the locale environment such as the characteristics of the soil and the climate.

The analysis of the genetic sequences of the bean that encode genes associated with symbiosis, revealed variation of NFR1, NFR5 and NIN over the representative accessions of the Mesoamerican, the Andean, and the AHD gene pools. It is proposed that a receptor complex composed of NFR1 and NFR5 initiates signal transduction in response to Nod-factor synthesized and released by rhizobia^27^. Although the variation consisted mainly in neutral-amino-acid substitutions, thus suggesting only minimal changes in the functionality, if any at all; we could cite the convincing and relevant evidence reported by Radutoiu et al*.*^27^ that the amino-acid residue 118 of the second LysM module of NFR5 is essential for the recognition of rhizobia by species of *Lotus japonicus* and *Lotus filicaulis*. Our finding that the Mesoamerican-bean NFR5 has glutamine (Q) in position 151, whereas the Andean and the AHD both have proline (P)—neither of which amino acids is neutral—would merit further investigation to evaluate if such a mutation might play a role in nodulation preference. Although this result must be considered with caution, we found that the conserved polymorphism in the NFR1 and NFR5 proteins has caused the beans representative of the genetic pool Ecuador-Peru—*i. e.*, the AHD—to be grouped in a cluster separate from those of Mesoamerica and the Andes. What we found to be interesting was that the phylogenetic and RMSD profiles of grouping the sequences are consistent with different evolutionary pathways in beans from the AHD and the Andean areas. This observation agrees with the proposal of Randón-Anaya et al*.*^2^ that those former beans from northern Peru-Ecuador originated from an ancestral form earlier than that of Mesoamerican- and Andean-bean genotypes. In addition, by applying a suppressive subtractive hybridization approach a set of bean genes were identified in our laboratory to be expressed in early step of infection by the cognate rhizobia^28^. Taken these results together, we conclude that genomic regions and patterns of expression in the host appear associated with an affinity for nodulation.

Within a broader context, we believe that our results on the biogeography of bean-rhizobia interactions in the region where the origin and domestication of the host plants occurred provide novel useful issues to be considered in inoculation programs, for instance those involving selection of strains and cultivars, and invite to validate these findings in follow up field trials.

## Methods

### Biological material

Table 1 describes the representative rhizobial strains and bean accessions used in this work.

Common bean seeds and permission for laboratory study were provided by the Centro Internacional de Agricultura Tropical (CIAT), Cali Colombia, with study protocol that comply with relevant institutional, national, and international guidelines and legislation.

### Coinoculation of bean

The experiments consisted in the parallel inoculation of plant representatives of the Mesoamerican, Ecuadorian, Peruvian, and Andean gene pools, with the same one-to-one mixture of rhizobial strains for inoculating each bean root. The incubation, nodule collection, and rhizobia isolation were done as previously described^9^. Briefly, cultures of *R. etli* strains showing *nod*C type α, type γ and type δ were grown separately to late exponential phase in yeast mannitol (YM) broth. Rhizobial mixtures of two strains having each one distinguishable *nod*C type (ratio 1:1) were made, and 10 ml of this mixture, containing 10^8^ bacteria, was applied per plant. Three independent coinoculation experiments were performed in which at least two plants of each accession were individually inoculated with a mixture of strains. Nodules were harvested 3 weeks after inoculation and the surface-disinfected nodules were quick-frozen in liquid nitrogen and thereafter kept at –80 °C until use for DNA extraction.

Bean plants were grown in 500 mL pots containing autoclaved perlite substrate, except in experiments to assess nodule occupancy in soil samples. Two contrasting soil types from Argentina were assayed with characteristics as it follows: A red clay soil (Ultisol) and a silty loam soil (Typic Argiudoll), containing respectively 3.80% and 3.02% organic matter, 1.9% and 1.5% oxidable carbon content, 0.20% and 0.16% total nitrogen, 8.1 and 2.9 ppm of extractable phosphorus, and pH 6.08 and 7.12.

### *nod*C-gene typing

After surface sterilization in 20% H_2_O_2_ and thoroughly washing in sterile water, nodules were individually crushed in 100 µl of sterile deionized water, and an aliquot sample of 1–5 µl used for the polymerase-chain-reaction. The PCR amplified *nod*C genes of rhizobia after *Hin*fI-endonuclease restriction was carried out on the rhizobial samples from individual nodules as described by Aguilar et al.^9,9^. Nodule occupancy was defined as the proportion of nodules occupied by each strain. Arcsine root square transformations of the values were used for mean comparisons. Statistical analyses were performed with nonparametric Kruskal–Wallis H test by using Infostat software version 1.0^29^.

### Analysis of bean genes

Genes previously reported to be associated with symbiotic nitrogen fixation were selected for a comparative analysis across the common bean genomes and the model legumes *Medicago truncatula*, *Lotus japonicus* and *Glycine max*. Sequences of five genes were examined namely gene RabA2, ASTRAY, NIN, NFR1 and NFR5. RabA2, is a gene coding for a small GTPase, associated with a major SNP for nodulation^12^; ASTRAY, associated to a QTL linked to nodulation^13^, is a transcription factor functional in nodulation regulation^14^, NIN is a transcription factor essential for nodulation^15^, and NFR1 and NFR5, receptor proteins for rhizobia nodulation factor^16^. The nucleotide sequences of the genes NFR1 and NFR5 of accessions G21244, G21245, G23587, and G23724 were retrieved from the Sequence-Reading-File Data, available at the server of the National Center for Biotechnology Information and at accession numbers listed in SI Table 1. The translation was done by applying the ExPASy Translate Tool (https://web.expasy.org/translate/). The protein sequences of G19833 were obtained from GenBank (accession numbers XP_007141617 and XP_007156886), whereas for BAT93 the identifiers corresponding to the two chromosomal sequences CM003679.1 and CM003673.1 were used.

Sequence alignments were obtained with the Clustal-X v2.1 software^30^ and the phylogenetic analysis performed by applying the Maximum Likelihood Estimation with the MEGA X tool^31^, substitution model Jones Taylor-Thornton. The phylogeny estimates were calculated by performing 1,000 bootstrap replicates.

Molecular modeling tools in the web site Swiss-model (https://swissmodel.expasy.org/) and the software Modeller v9.25^32^ were used to generate models of NFR1 and NFR5 that were evaluated by applying PROCHECK (http://www.ebi.ac.uk/thornton-srv/databases/pdbum/). PyMOL (Molecular Graphics System v2.4, Schrödinger, LLC) and VMD^32^ were used to assess topology and calculate values of root-mean-square deviation, RMSD.

## Supplementary Information

Supplementary Information.
