# Supplementary material for: Nodulation competitiveness and diversification of symbiosis genes in common beans from the American centers of domestication
Source: Sci Rep. 2022 Mar 17;12:4591. doi: 10.1038/s41598-022-08720-0 (PMC8931114; doi:10.1038/s41598-022-08720-0)
Supplement: Supplementary file 1 — Supplementary Information. [file 41598_2022_8720_MOESM1_ESM.pdf]

NODULATION COMPETITIVENESS AND DIVERSIFICATION OF SYMBIOSIS GENES IN COMMON BEANS FROM THE AMERICAN CENTERS OF DOMESTICATION

O. Mario Aguilar, Mónica M. Collavino and Ulises Mancini

Supplementary Material

SI. Fig. 1: Protein alignment of *P. vulgaris* gene products Nfr1 (A), Nfr5 (B), Astray (C), RabA2 (D) and NIN (E), by using the Clustal-X v2.1 software [30]. Red color represents highly conserved residues; blue color represents variants in sequence.

1.A Nfr1 protein alignment

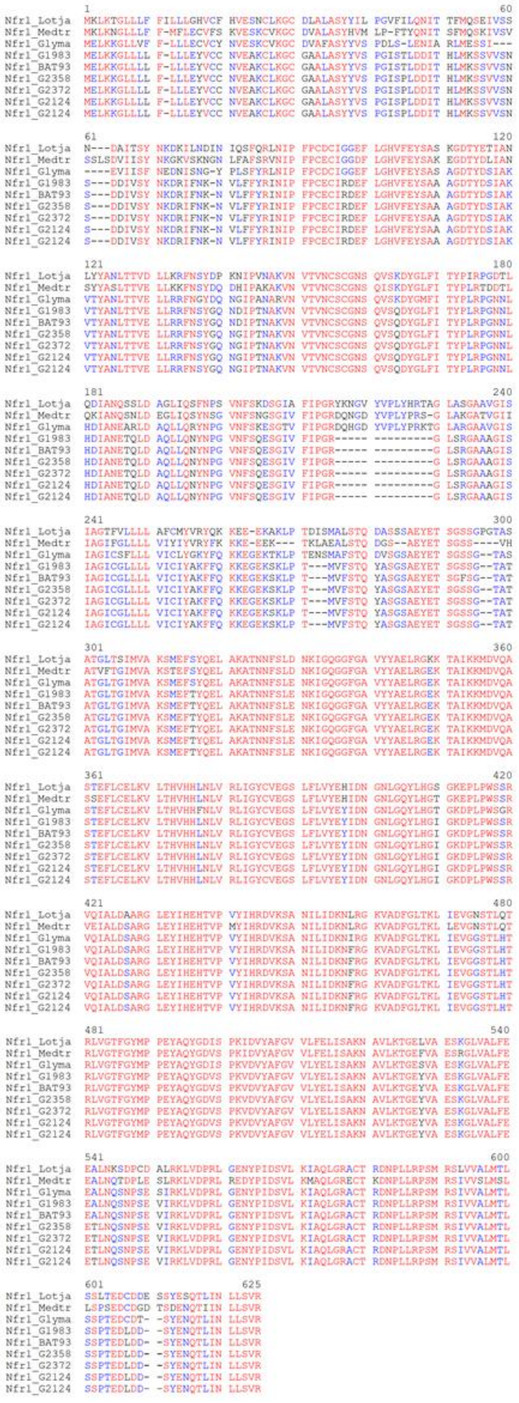

1.B Nfr5 protein alignment

```
             1                                                            60
Nfr5_Lotja   MAVFF--LTS GSLSLFLALT LLF-TNIAAR SEKISGPDFS CPVDSPPSCE TYVTYTAQSP
Nfr5_Glyma   MAVFFPFLPL HSQILCLVIM LFS-TNIVAQ SQQDNRTNFS CPSDSPPSCE TYVTYIAQSP
Nfr5_G1983   MAVFFVSLTL GAQILYVVLM -FF-TCIEAQ SQQTNGTNFS CPSNSPPSCE TYVTYISQSP
Nfr5_BAT93   MAVFFVSLTL GAQILYVVLM -FF-TCIEAQ SQQTNGTNFS CPSNSPPSCE TYVTYISQSP
Nfr5_G2358   MAVFFVSLTL GAQILYVVLM -FS-TCIEAQ SQQTNGTNFS CPSNSPPSCE TYVTYITQSP
Nfr5_G2372   MAVFFVSLTL GAQILYVVLM -FS-TCIEAQ SQQTNGTNFS CPSNSPPSCE TYVTYITQSP
Nfr5_G2124   MAVFFVSLTL GAQILYVVLM -FS-TCIEAQ SQQTNGTNFS CPSNSPPSCE TYVTYITQSP
Nfr5_G2124   MAVFFVSLTL GAQILYVVLM -FS-TCIEAQ SQQTNGTNFS CPSNSPPSCE TYVTYITQSP
Nfr5_Medtr   MSAFF--LPS SSHALFLVLM LFFLTNISAQ PLYISETNFT CPVDSPPSCE TYVAYRAQSP

             61                                                          120
Nfr5_Lotja   NLLSLTNISD IFDISPLSIA RASNIDAGKD KLVPGQVLLV PVTCGCAGNH SSANTSYQIQ
Nfr5_Glyma   NFLSLTNISN IFDTSPLSIA RASNLEPMDD KLVKDQVLLV PVTCGCTGNR SFANISYEIN
Nfr5_G1983   NFLSLTSVSN IFDTSPLSIA RASNLQHEED KLIPGQVLLI PVTCGCTGNR SFANISYEIN
Nfr5_BAT93   NFLSLTSVSN IFDTSPLSIA RASNLQHEED KLIPGQVLLI PVTCGCTGNR SFANISYEIN
Nfr5_G2358   NFLSLTSVSN IFDTSPLSIA RASNLQHEED KLIPGQVLLI PVTCGCTGNR SFANISYEIN
Nfr5_G2372   NFLSLTSVSN IFDTSPLSIA RASNLQHEED KLIPGQVLLI PVTCGCTGNR SFANISYEIN
Nfr5_G2124   NFLSLTSVSN IFDTSPLSIA RASNLQHEED KLIPGQVLLI PVTCGCTGNR SFANISYEIN
Nfr5_G2124   NFLSLTSVSN IFDTSPLSIA RASNLQHEED KLIPGQVLLI PVTCGCTGNR SFANISYEIN
Nfr5_Medtr   NFLSLSNISD IFNLSPLRIA KASNIEAEDK KLIPDQLLLV PVTCGCTKNH SFANITYSIK

             121                                                         180
Nfr5_Lotja   LGDSYDFVAT TLYENLTNWN IVQASNPGVN PYLLPERVKV VFPLFCRCPS KNQLNKGIQY
Nfr5_Glyma   QGDSFYFVAT TSYENLTNWR AVMDLNPVLS PNKLPIGIQV VFPLFCKCPS KNQLDKEIKY
Nfr5_G1983   QGDSFYFVAT TLYQNLTNWH AVMDLNPGLS PFTLPIGIQV VIPLFCKCPS KNQLDRGIKY
Nfr5_BAT93   QGDSFYFVAT TLYQNLTNWH AVMDLNPGLS QFTLPIGIQV VIPLFCKCPS KNQLDRGIKY
Nfr5_G2358   QGDSFYFVAT TLYQNLTNWH AVMDLNPGLS PFTLPIGIQV VIPLFCKCPS KNQLDRGIKY
Nfr5_G2372   QGDSFYFVAT TLYQNLTNWH AVMDLNPGLS PFTLPIGIQV VIPLFCKCPS KNQLDRGIKY
Nfr5_G2124   QGDSFYFVAT TLYQNLTNWH AVMDLNPGLS PFTLPIGIQV VIPLFCKCPS KNQLDRGIKY
Nfr5_G2124   QGDSFYFVAT TLYQNLTNWH AVMDLNPGLS PFTLPIGIQV VIPLFCKCPS KNQLDRGIKY
Nfr5_Medtr   QGDNFFILSI TSYQNLTNYL EFKNFNPNLS PTLLPLDTKV SVPLFCKCPS KNQLNKGIKY

             181                                                         240
Nfr5_Lotja   LITYVWKPND NVSLVSAKFG ASPADILTEN RYGQDFTAAT NLPILIPVTQ LPELTQPSSN
Nfr5_Glyma   LITYVWKPGD NVSLVSDKFG ASPEDIMSEN NYGQNFTAAN NLPVLIPVTR LPVLARSPSD
Nfr5_G1983   LITHVWQPND NVSFVSNKLG ASPQDILSEN NYGQNFTAAS NLPVLIPVTL LPDLIQSPSD
Nfr5_BAT93   LITHVWQPND NVSFVSNKLG ASPQDILSEN NYGQNFTAAS NLPVLIPVTL LPDLIQSPSD
Nfr5_G2358   LITHVWQPND NVSFVSNKLG ASPQDILSEN NYGQNFTAAS NLPVLIPVTL LPDLIQSPSD
Nfr5_G2372   LITHVWQPND NVSFVSNKLG ASPQDILSEN NYGQNFTAAS NLPVLIPVTL LPDLIQSPSD
Nfr5_G2124   LITHVWQPND NVSFVSNKLG ASPQDILSEN NYGQNFTAAS NLPVLIPVTL LPDLIQSPSD
Nfr5_G2124   LITHVWQPND NVSFVSNKLG ASPQDILSEN NYGQNFTAAS NLPVLIPVTL LPDLIQSPSD
Nfr5_Medtr   LITYVWQDND NVTLVSSKFG ASQVEMLAEN NH--NFTAST NRSVLIPVTS LPKLDQPSSN

             241                                                         300
Nfr5_Lotja   GRKSSI-HLL VILGITLGCT LLTAVLTGTL VYVYCRRKKA LNRTASSAET ADKLLSGVSG
Nfr5_Glyma   GRKGGI-RLP VIIGISLGCT LLVLVLAVLL VYVYCLKMKT LNRSASSAET ADKLLSGVSG
Nfr5_G1983   GRKHRI-GLP VIIGISLGCT LLVVVSAILL VCVCCLKMKS LNRSASSAET ADKLLSGVSG
Nfr5_BAT93   GRKHRI-GLP VIIGISLGCT LLVVVSAILL VCVCCLKMKS LNRSASSAET ADKLLSGVSG
Nfr5_G2358   GRKHRI-GLP VIIGISLGCT LLVVVSAILL VCVCCLKMKS LNRSASSAET ADKLLSGVSG
Nfr5_G2372   GRKHRI-GLP VIIGISLGCT LLVVVSAILL VCVCCLKMKS LNRSASSAET ADKLLSGVSG
Nfr5_G2124   GRKHRI-GLP VIIGISLGCT LLVVVSAILL VCVCCLKMKS LNRSASSAET ADKLLSGVSG
Nfr5_G2124   GRKHRI-GLP VIIGISLGCT LLVVVSAILL VCVCCLKMKS LNRSASSAET ADKLLSGVSG
Nfr5_Medtr   GRKSSSQNLA LIIGISLGSA FFILVLTLSL VYVYCLKMKR LNRSTSSSET ADKLLSGVSG

             301                                                         360
Nfr5_Lotja   YVSKPNVYEI DEIMEATKDF SDECKVGESV YKANIEGRVV AVKKIKEGGA NEELKILQKV
Nfr5_Glyma   YVSKPTMYET DAIMEATMNL SEQCKIGESV YKANIEGKVL AVKRFKED-V TEELKILQKV
Nfr5_G1983   YVSKPTMYET GAILEATMNL SEQCKIGESV YKANIEGKVL AVKRFKED-V TEELKILQKV
Nfr5_BAT93   YVSKPTMYET GAILEATMNL SEQCKIGESV YKANIEGKVL AVKRFKED-V TEELKILQKV
Nfr5_G2358   YVSKPTMYET GAILEATMNL SEQCKIGESV YKANIEGKVL AVKRFKED-V TEELKILQKV
Nfr5_G2372   YVSKPTMYET GAILEATMNL SEQCKIGESV YKANIEGKVL AVKRFKED-V TEELKILQKV
Nfr5_G2124   YVSKPTMYET GAILEATMNL SEQCKIGESV YKANIEGKVL AVKRFKED-V TEELKILQKV
Nfr5_G2124   YVSKPTMYET GAILEATMNL SEQCKIGESV YKANIEGKVL AVKRFKED-V TEELKILQKV
Nfr5_Medtr   YVSKPTMYEI DAIMEGTTNL SDNCKIGESV YKANIDGRVL AVKKIKKD-A SEELKILQKV

             361                                                         420
Nfr5_Lotja   NHGNLVKLMG VSSGYDGNCF LVYEYAENGS LAEWLFSKS- ---SGTPNSL TWSQRISIAV
Nfr5_Glyma   NHGNLVKLMG VSSDNDGNCF VVYEYAENGS LDEWLFSKSC SDTSNSRASL TWCQRISMAV
Nfr5_G1983   NHGNLVKLMG VSSDNDGNCF VVYEYAENGS LQEWLFAKSC SETLNSRTSL TWCQRISIAV
Nfr5_BAT93   NHGNLVKLMG VSSDNDGNCF VVYEYAENGS LEEWLFAKSC SETSNSRTSL TWCQRISIAV
Nfr5_G2358   NHGNLVKLMG VSSDNDGNCF VVYEYAENGS LQGWLFAKSC SETSNSRTSL TWCQRISIAV
Nfr5_G2372   NHGNLVKLMG VSSDNDGNCF VVYEYAENGS LEGWLFAKSC SETSNSRTSL TWCQRISIAV
Nfr5_G2124   NHGNLVKLMG VSSDNDGNCF VVYEYAENGS LQGWLFAKSC SETSNSRTSL TWCQRISIAV
Nfr5_G2124   NHGNLVKLMG VSSDNDGNCF VVYEYAENGS LEEWLFAKSC SETSNSRTSL TWCQRISIAV
Nfr5_Medtr   NHGNLVKLMG VSSDNDGNCF LVYEYAENGS LEEWLFSES- SKTSNSVVSL TWSQRITIAM

             421                                                         480
Nfr5_Lotja   DVAVGLQYMH EHTYPRIIHR DITTSNILLD SNFKAKIANF AMARTSTNPM MPKIDVFAFG
Nfr5_Glyma   DVAMGLQYMH EHAYPRIVHR DITSSNILLD SNFKAKIANF SMARTFTNPM MPKIDVFAFG
Nfr5_G1983   DVSMGLQYMH EHAYPRIVHR DITSSNILLD SNFKAKIANF SMARTFTNPM MSKIDVFAFG
Nfr5_BAT93   DVSMGLQYMH EHAYPRIVHR DITSSNILLD SNFKAKIANF SMARTFTNPM MSKIDVFAFG
Nfr5_G2358   DVSMGLQYMH EHAYPRIVHR DITSSNILLD SNFKAKIANF SMARTFTNPM MSKIDVFAFG
Nfr5_G2372   DVSMGLQYMH EHAYPRIVHR DITSCNILLD SNFKAKIANF SMARTFTNPM MSKIDVFAFG
Nfr5_G2124   DVSMGLQYMH EHAYPRIVHR DITSCNILLD SNFKAKIANF SMARTFTNPM MSKIDVFAFG
Nfr5_G2124   DVSMGLQYMH EHAYPRIVHR DITSCNILLD SNFKAKIANF SMARTFTNPM MSKIDVFAFG
Nfr5_Medtr   DVAIGLQYMH EHTYPRIIHR DITTSNILLG SNFKAKIANF GMARTSTNSM MPKIDVFAFG

             481                                                         540
Nfr5_Lotja   VLLIELLTGR KAMTTKENGE VVMLWKDMWE IFDIEENREE RIRKWMDPNL ESFYHIDNAL
Nfr5_Glyma   VVLIELLTGR KAMTTKENGE VVMLWKDIWK IFDQEENREE RLKKWMDPKL ESYYPIDYAL
Nfr5_G1983   VVLIELLTGR KAMTTKENGE VVMLWTDIWK IFDQEENREE RLRKWMDPKL DNYYPIDYAL
Nfr5_BAT93   VVLIELLTGR KAMTTKENGE VVMLWKDIWK IFDQEENREE RLRKWMDPKL DNYYPIDYAL
Nfr5_G2358   VVLIELLTGR KAMTTKENGE VVMLWKDIWK IFDQEENREE RLRKWMDPKL DNYYPIDYAL
Nfr5_G2372   VVLIELLTGR KAMTTKENGE VVMLWKDIWK IFDQEENREE RLRKWMDPKL DNYYPIDYAL
Nfr5_G2124   VVLIELLTGR KAMTTKENGE VVMLWKDIWK IFDQEENREE RLRKWMDPKL DNYYPIDYAL
Nfr5_G2124   VVLIELLTGR KAMTTKENGE VVMLWTDIWK IFDQEENREE RLRKWMDPKL DNYYPIDYAL
Nfr5_Medtr   VVLIELLTGK KAMTTKENGE VVILWKDFWK IFDLEGNREE RLRKWMDPKL ESFYPIDNAL

             541                                                         600
Nfr5_Lotja   SLASLAVNCT ADKSLSRPSM AEIVLSLSFL TQQSSNPTLE RSLTSSGLDV EDDAHIVTSI
Nfr5_Glyma   SLASLAVNCT ADKSLSRPTI AEIVLSLSLL TQPSP-ATLE RSLTSSGLDV EA-TQIVTSI
Nfr5_G1983   SLASLAMNCT ADKSLSRPTI AEIVLSLSLL TQPSP-ATLE RSLTSSGLDV EA-TQIVTSI
Nfr5_BAT93   SLASLAVNCT ADKSLSRPTI AEIVLSLSLL TQPSP-ATLE RSLTSSGLDV EA-TQIVTSI
Nfr5_G2358   SLASLAVNCT ADKSLSRPTI AEIVLSLSLL TQPSP-GTLE RSLTSSGLDV EA-TQIVTSI
Nfr5_G2372   SLASLAVNCT ADKSLSRPTI AEIVLSLSLL TQPSP-GTLE RSLTSSGLDV EA-TQIVTSI
Nfr5_G2124   SLASLAVNCT ADKSLSRPTI AEIVLSLSLL TQPSP-GTLE RSLTSSGLDV EA-TQIVTSI
Nfr5_G2124   SLASLAVNCT ADKSLSRPTI AEIVLSLSLL TQPSP-GTLE RSLTSSGLDV EA-TQIVTSI
Nfr5_Medtr   SLASLAVNCT ADKSLSRPTI AEIVLCLSLL NQPSSEPMLE RSLTS-GLDA EA-THVVTSV

             601
Nfr5_Lotja   TAR
Nfr5_Glyma   AAR
Nfr5_G1983   SAR
Nfr5_BAT93   SAR
Nfr5_G2358   SAR
Nfr5_G2372   SAR
Nfr5_G2124   SAR
Nfr5_G2124   SAR
Nfr5_Medtr   IAR
```

1.C Astray protein alignment

```
             1                                                           60
ASTR_Lotja   MDQHGGVATG SHERNELVRV RHG-SDHGSK PVKNLNGQIC QICGETVGLT ANGDIFVACH
ASTR_Medtru  ME---GVETG SHERNELVRA RHDVSETGSK PLKNLNGQIC QICGDTVGLT ATGEVFVACH
ASTR_Glymax  MERSGGMVTG SHERNELVRV RHG-SDSGSK PLKNLNGQIC QICGDTIGLT ATGDLFVACH
ASTR_G19833  MERSGGMVTG SHERNELVTV RHG-SDSGSK PVKNLNGQIC QICGDTIGLT ATGDVFVACH
ASTR_BAT93   MERSGGMVTG SHERNELVTV RHG-SDSGSK PVKNLNGQIC QICGDTIGLT ATGDVFVACH
ASTR_G23587  MERSGGMVTG SHERNELVTV RHG-SDSGSK PVKNLNGQIC QICGDTIGLT ATGDVFVACH
ASTR_G23724  MERSGGMVTG SHERNELVTV RHG-SDSGSK PVKNLNGQIC QICGDTIGLT ATGDVFVACH
ASTR_G21244  MERSGGMVTG SHERNELVTV RHG-SDSGSK PVKNLNGQIC QICGDTIGLT ATGDVFVACH
ASTR_G21245  MERSGGMVTG SHERNELVTV RHG-SDSGSK PVKNLNGQIC QICGDTIGLT ATGDVFVACH

             61                                                         120
ASTR_Lotja   ECAFQLCHPC YEYERNNVSP YCPQCKTRYE SHKESSQMEG DDDDDDDEDD VDDLENEVKY
ASTR_Medtru  ECSFPLCYPC YEYEWKNVIQ SCPQCKTRFK SHKDDPRVKG GDD----EDD VDDLRNEVKY
ASTR_Glymax  ECGFPLCHSC YEYELKNVSQ SCPQCKTTFT SRQEGAEVEG DDDD---EDD ADDLDNGINY
ASTR_G19833  ECGLPLCHSC YEYELKNVSQ SCPQCKTRFT SHQEGAGVEG DDDD---DED ADDLDNEINY
ASTR_BAT93   ECGFPLCHSC YEYELKNVSQ SCPQCKTRFT SHQEGAGVEG DDDD---DED ADDLDNEINY
ASTR_G23587  ECGFPLCHSC YEYELKNVSQ SCPQCKTRFT SHQEGAGVEG DDDD---DED ADDLDNEINY
ASTR_G23724  ECGFPLCHSC YEYELKNVSQ SCPQCKTRFT SHQEGAGVEG DDDD---DED ADDLDNEINY
ASTR_G21244  ECGFPLCHSC YEYELKNVSQ SCPQCKTRFT SHQEGAGVEG DDDD---DED ADDLDNEINY
ASTR_G21245  ECGFPLCHSC YEYELKNVSQ SCPQCKTRFT SHQEGAGVEG DDDD---DED ADDLDNEINY

             121                                                        180
ASTR_Lotja   GQGNRMKARL QWEEDADLSS SSGHDSQMQN PHLTNGQLMS GENLCAT--- -----TSGPM
ASTR_Medtru  GQGNNAKAGW QWDEDADLSS SSGHDSQLQN PHLTNGQLMS GDIPCATSDT QSVQTTSGPL
ASTR_Glymax  GQGNNSKSGM LWEEDADLSS SSGHDSHIPN PHLVNGQPMS GEFPCATSDA QSMQTTSDPM
ASTR_G19833  GQGNNSKAGM QWEEDADFSS SSGHDSQIPN PHLANGQPMS GDIPCATSDA QSMQTTSGLM
ASTR_BAT93   GQGNNSKAGM QWEEDADFSS SSGHDSQIPN PHLANGQPMS GDIPCATSDA QSMQTTSGLM
ASTR_G23587  GQGNNSKAGM QWEEDADFSS SSGHDSQIPN PHLANGQPMS GDIPCATSDA QSMQTTSGLM
ASTR_G23724  GQGNNSKAGM QWEEDADFSS SSGHDSQIPN PHLANGQPMS GDIPCATSDA QSMQTTSGLM
ASTR_G21244  GQGNNSKAGM QWEEDADFSS SSGHDSQIPN PHLANGQPMS GDIPCATSDA QSMQTTSGLM
ASTR_G21245  GQGNNSKAGM QWEEDADFSS SSGHDSQIPN PHLANGQPMS GDIPCATSDA QSMQTTSGLM

             181                                                        240
ASTR_Lotja   GQSKKVQSLP YVDPKQPGLE SDEEIRRVPE FGGESAATSA SRPDTGSNPG PERVQGAGEG
ASTR_Medtru  GQSENVHSRA YVDPKQPGLE SDEEIRRVPD MGGESAGTSA SRKGTGSTAG PERVQGAGEG
ASTR_Glymax  GQSEKVHSLP YADPKQPGPE SDEEIRRVPE IGGESAGTSA SRPDAGSNAG TERAQGTGDS
ASTR_G19833  ---------- -AAAKQPGPE SDEEIRRVPE IGGESAGTVA SRPDARSNAG AERVQGTGEG
ASTR_BAT93   ---------- -AAAKQPGPE SDEEIRRVPE IGGESAGTVA SRPDARSNAG AERVQGTGEG
ASTR_G23587  ---------- -AAAKQPGPE SDEEIRRVPE IGGESAGTVA SRPDARSNAG AERVQGTGEG
ASTR_G23724  ---------- -AAAKQPGPE SDEEIRRVPE IGGESAGTVA SRPDARSNAG AERVQGTGEG
ASTR_G21244  ---------- -AAAKQPGPE SDEEIRRVPE IGGESAGTVA SRPDARSNAG AERVQGTGEG
ASTR_G21245  ---------- -AAAKQPGPE SDEEIRRVPE IGGESAGTVA SRPDARSNAG AERVQGTGEG

             241                                                        300
ASTR_Lotja   QKKRGRSSAD KESKRLKRLL RNRVSAQQAR ERKKAYLTDL ETKVKDLETN NSELKERLST
ASTR_Medtru  QKKRGRSPAD KESKRLKRLL RNRVSAQQAR ERKKAYLSDL ETRVNDLEKK NSELKEKLST
ASTR_Glymax  QKKRGRSPAD KESKRLKRLL RNRVSAQQAR ERKKAYLIDL ETRVKDLEKK NSELKERLST
ASTR_G19833  QKKRGRSPAD KESKRLKRLL RNRVSAQQAR ERKKAYLIDL ETRVKDLEKK NSELKERLST
ASTR_BAT93   QKKRGRSPAD KESKRLKRLL RNRVSAQQAR ERKKAYLIDL ETRVKDLEKK NSELKERLST
ASTR_G23587  QKKRGRSPAD KESKRLKRLL RNRVSAQQAR ERKKAYLIDL ETRVKDLEKK NSELKERLST
ASTR_G23724  QKKRGRSPAD KESKRLKRLL RNRVSAQQAR ERKKAYLIDL ETRVKDLEKK NSELKERLST
ASTR_G21244  QKKRGRSPAD KESKRLKRLL RNRVSAQQAR ERKKAYLIDL ETRVKDLEKK NSELKERLST
ASTR_G21245  QKKRGRSPAD KESKRLKRLL RNRVSAQQAR ERKKAYLIDL ETRVKDLEKK NSELKERLST

             301                  330
ASTR_Lotja   LQNENQMLRQ ILKNTTASRR GSNGGTNNAE
ASTR_Medtru  LQNENQMLRQ ILKNTTASRR GSNGGSNTAE
ASTR_Glymax  LQNENQMLRQ ILKNTTASRR GSNSGTNNAE
ASTR_G19833  LQNENQMLRQ ILKNTTASRR GSNSGTNNAE
ASTR_BAT93   LQNENQMLRQ ILKNTTASRR GSNSGTNNAE
ASTR_G23587  LQNENQMLRQ ILKNTTASRR GSNSGTNNAE
ASTR_G23724  LQNENQMLRQ ILKNTTASRR GSNSGTNNAE
ASTR_G21244  LQNENQMLRQ ILKNTTASRR GSNSGTNNAE
ASTR_G21245  LQNENQMLRQ ILKNTTASRR GSNSGTNNAE
```

1.D Rab protein alignment

```
              1                                                            60
Rab_Glyma     MAHRVDHEYD YLFKIVLIGD SGVGKSNILS RFTRNEFCLE SKSTIGVEFA TRTLQVEGKT
Rab_19833     MTHRVDHEYD YLFKIVLIGD SGVGKSNILS RFTRNEFCLE SKSTIGVEFA TRTLQVEGKT
Rab_BAT93     MTHRVDHEYD YLFKIVLIGD SGVGKSNILS RFTRNEFCLE SKSTIGVEFA TRTLQVEGKT
Rab_21244     MTHRVDHEYD YLFKIVLIGD SGVGKSNILS RFTRNEFCLE SKSTIGVEFA TRTLQVEGKT
Rab_23724     MTHRVDHEYD YLFKIVLIGD SGVGKSNILS RFTRNEFCLE SKSTIGVEFA TRTLQVEGKT
Rab_23587     MTHRVDHEYD YLFKIVLIGD SGVGKSNILS RFTRNEFCLE SKSTIGVEFA TRTLQVEGKT
Rab_21245     MTHRVDHEYD YLFKIVLIGD SGVGKSNILS RFTRNEFCLE SKSTIGVEFA TRTLQVEGKT
Rab_Lotja     MAHRVDHEYD YLFKIVLIGD SGVGKSNILS RFTRNEFCLE SKSTIGVEFA TRTLQVEGKT
Rab_Medtr     MAHRVDHEYD YLFKIVLIGD SGVGKSNILS RFTRNEFCLE SKSTIGVEFA TRTLQVEGKT

              61                                                          120
Rab_Glyma     VKAQIWDTAG QERYRAITSA YYRGAVGALL VYDITKRQTF DNVQRWLREL RDHADSNIVI
Rab_19833     VKAQIWDTAG QERYRAITSA YYRGAVGALL VYDITKRQTF DNVQRWLREL RDHADSNIVI
Rab_BAT93     VKAQIWDTAG QERYRAITSA YYRGAVGALL VYDITKRQTF DNVQRWLREL RDHADSNIVI
Rab_21244     VKAQIWDTAG QERYRAITSA YYRGAVGALL VYDITKRQTF DNVQRWLREL RDHADSNIVI
Rab_23724     VKAQIWDTAG QERYRAITSA YYRGAVGALL VYDITKRQTF DNVQRWLREL RDHADSNIVI
Rab_23587     VKAQIWDTAG QERYRAITSA YYRGAVGALL VYDITKRQTF DNVQRWLREL RDHADSNIVI
Rab_21245     VKAQIWDTAG QERYRAITSA YYRGAVGALL VYDITKRQTF DNVQRWLREL RDHADSNIVI
Rab_Lotja     VKAQIWDTAG QERYRAITSA YYRGAVGALL VYDITKRQTF DNVQRWLREL RDHADSNIVI
Rab_Medtr     VKAQIWDTAG QERYRAITSA YYRGAVGALL VYDITKRQTF DNVNRWLREL RDHADSNIVI

              121                                                         180
Rab_Glyma     MMAGNKSDLS HLRAVSEDDG QALAEREGLS FLETSALEAT NIEKAFQTIL TEIYHIVSKK
Rab_19833     MMAGNKSDLS HLRAVYEDDG QNLAEREGLS FLETSALEAT NIEKAFQTIL TEIYHIVSKK
Rab_BAT93     MMAGNKSDLS HLRAVYEDDG QNLAEREGLS FLETSALEAT NIEKAFQTIL TEIYHIVSKK
Rab_21244     MMAGNKSDLS HLRAVYEDDG QNLAEREGLS FLETSALEAT NIEKAFQTIL TEIYHIVSKK
Rab_23724     MMAGNKSDLS HLRAVYEDDG QNLAEREGLS FLETSALEAT NIEKAFQTIL TEIYHIVSKK
Rab_23587     MMAGNKSDLS HLRAVYEDDG QNLAEREGLS FLETSALEAT NIEKAFQTIL TEIYHIVSKK
Rab_21245     MMAGNKSDLS HLRAVYEDDG QNLAEREGLS FLETSALEAT NIEKAFQTIL TEIYHIVSKK
Rab_Lotja     MMAGNKSDLN HLRAVSEDDG GALSEKEGLS FLETSALEAT NIEKAFQTIL TEIYHIVSKK
Rab_Medtr     MMAGNKSDLN HLRAVSEDDG QALAEKEGLS FLETSALEAT NIEKAFQTIL TEIYHIVSKK

              181                               216
Rab_Glyma     ALAAQEAAVG TTLPGQGTTI NVGDASGNTK RGCCST
Rab_19833     ALAAQEAGVG TTLPGQGTTI NVGDASGNTK RGCCST
Rab_BAT93     ALAAQEAGVG TTLPGQGTTI NVGDASGNTK RGCCST
Rab_21244     ALAAQEAGVG TTLPGQGTTI NVGDASGNTK RGCCST
Rab_23724     ALAAQEAGVG TTLPGQGTTI NVGDASGNTK RGCCST
Rab_23587     ALAAQEAGVG TTLPGQGTTI NVGDASGNTK RGCCST
Rab_21245     ALAAQEAGVG TTLPGQGTTI NVGDASGNTK RGCCST
Rab_Lotja     ALAAQEATAG ASVPGQGTTI NVADTSGNTK KGCCST
Rab_Medtr     ALAAQEA-AG TSLPGQGTTI NVADSSANTK RGCCST
```

1.E NIN protein alignment

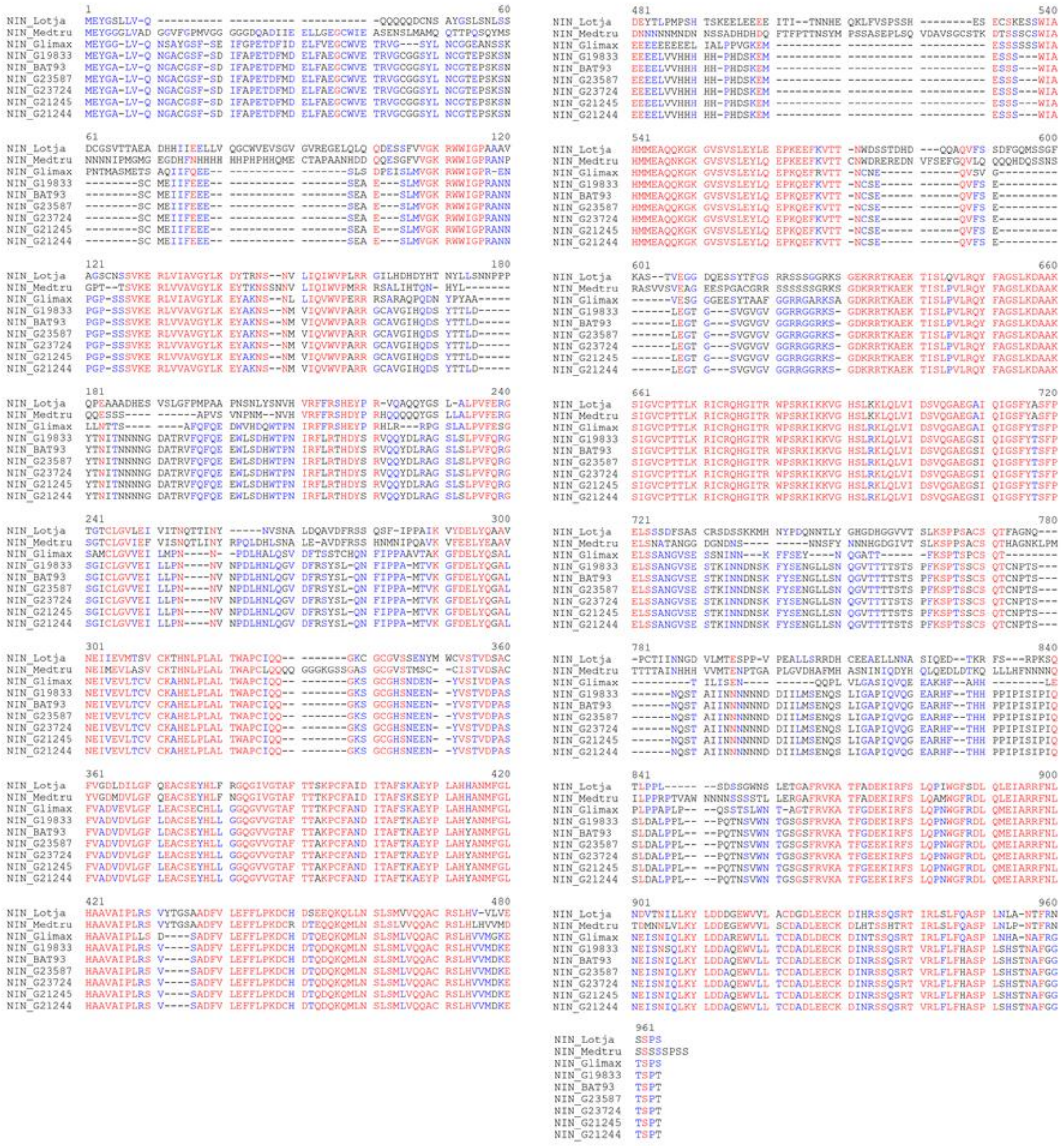

SI. Table 1: Accession number of sequences retrieved from data bases described in Methods that were used for the protein alignment and phylogenetic analysis

	**NFR1**	**NFR5**	**ASTRAY**	**NIN**	**RAB**
P. vul. G19833	XP_007141617	XP_007156886	XP_007146305	XP_007137300	ACV82919
P. vul. BAT93*	GCA_001517995	GCA_001517995	GCA_001517995	GCA_001517995	GCA_001517995
P. vul. G23587**	SRX2537723	SRX2537723	SRX2537723	SRX2537723	SRX2537723
P. vul. G23724**	SRX2537724	SRX2537724	SRX2537724	SRX2537724	SRX2537724
P. vul. G21245**	SRX2537725	SRX2537725	SRX2537725	SRX2537725	SRX2537725
P. vul. G21244**	SRX540119	SRX540119	SRX540119	SRX540119	SRX540119
Glycine max	XP_006575588	NP_001341125	NP_001237272	XP_003523488	XP_003537971
Lotus japonicus	CAE02591	CAE02598	BAC20318	CAB61243	CAA98179
Medicago trunc.	XP_024640165	XP_003611889	XP_013459310	XP_003618108	XP_013456161

*Identifier has not been assigned for BAT93 proteins and therefore they were inferred from its assembly accession.

**Idem for *P. vulgaris* genomes G23587, G23724, G21244 and G21245, identifier has not been yet assigned for proteins and therefore these were inferred from its SRAs accessions.

SI. Table 2: Analysis of model topology of proteins Nfr1 and Nfr5. PyMOL (Molecular Graphics System v2.4, Schrödinger, LLC) and VMD were used to assess topology and calculate values of root-mean-square deviation, RMSD [32, 33]

GENOME	PROTEIN	PDB ID	METHOD	RANGE	SEQUENCE IDENTITY	SEQUENCE SIMILARITY	COVERAGE	RESOLUTION	TEMPLATE	DESCRIPTION	RMSD
BAT93	nfr1	6CTH	Ray-X	289-580	36.71	0.38	0.48	1.70Å	Crystal Structure of Pathogenesis-related Protein 1G (PR-1G) Kinase Domain from Cacao	Concanavalin A-like lectin protein kinase family protein	0.000
G19833	nfr1	6CTH	Ray-X	289-580	36.71	0.38	0.48	1.70Å	Crystal Structure of Pathogenesis-related Protein 1G (PR-1G) Kinase Domain from Cacao	Concanavalin A-like lectin protein kinase family protein	0.000
G21244	nfr1	6CTH	Ray-X	289-580	37.06	0.38	0.48	1.70Å	Crystal Structure of Pathogenesis-related Protein 1G (PR-1G) Kinase Domain from Cacao	Concanavalin A-like lectin protein kinase family protein	1.104
G21245	nfr1	6CTH	Ray-X	289-580	37.06	0.38	0.48	1.70Å	Crystal Structure of Pathogenesis-related Protein 1G (PR-1G) Kinase Domain from Cacao	Concanavalin A-like lectin protein kinase family protein	1.104
G23587	nfr1	6CTH	Ray-X	289-580	37.06	0.38	0.48	1.70Å	Crystal Structure of Pathogenesis-related Protein 1G (PR-1G) Kinase Domain from Cacao	Concanavalin A-like lectin protein kinase family protein	1.104
G23724	nfr1	6CTH	Ray-X	289-580	37.06	0.38	0.48	1.70Å	Crystal Structure of Pathogenesis-related Protein 1G (PR-1G) Kinase Domain from Cacao	Concanavalin A-like lectin protein kinase family protein	1.104
BAT93	nfr5	6BFN	Ray-X	300-570	31.92	0.36	0.44	2.26Å	Crystal structure of human IRAK1	Interleukin-1 receptor-associated kinase 1 Crystal structure of human IRAK1	0.000
G19833	nfr5	6BFN	Ray-X	300-570	31.03	0.36	0.44	2.26Å	Crystal structure of human IRAK1	Interleukin-1 receptor-associated kinase 1 Crystal structure of human IRAK1	0.015
G21244	nfr5	6BFN	Ray-X	300-570	31.03	0.36	0.44	2.26Å	Crystal structure of human IRAK1	Interleukin-1 receptor-associated kinase 1 Crystal structure of human IRAK1	0.762
G21245	nfr5	6BFN	Ray-X	300-570	31.03	0.36	0.44	2.26Å	Crystal structure of human IRAK1	Interleukin-1 receptor-associated kinase 1 Crystal structure of human IRAK1	0.791
G23587	nfr5	6BFN	Ray-X	300-570	31.54	0.36	0.44	2.26Å	Crystal structure of human IRAK1	Interleukin-1 receptor-associated kinase 1 Crystal structure of human IRAK1	0.761
G23724	nfr5	6BFN	Ray-X	300-570	31.42	0.36	0.44	2.26Å	Crystal structure of human IRAK1	Interleukin-1 receptor-associated kinase 1 Crystal structure of human IRAK1	0.820
